# Triceps Innervation Pattern: Implications for Triceps Nerve to Deltoid Nerve Transfer

**DOI:** 10.1155/2013/132954

**Published:** 2013-12-30

**Authors:** Obaid Al-Meshal, Alain Gilbert

**Affiliations:** ^1^King Fahad National Guard Hospital, King Abdulaziz Medical City (KAMC), P.O. Box 22490, Mail Code 1446, Riyadh 11426, Saudi Arabia; ^2^Institut de la Main, Clinique Jouvenet, Paris, France

## Abstract

There are multiple nerve branches supplying the triceps. Traditionally, the nerve to the long head of triceps is utilized for nerve transfer to neurotize the deltoid muscle in patients with brachial plexus injuries. However, no anatomical studies were done to investigate which triceps nerve would be preferred for nerve transfer. This anatomical study was carried out to describe the innervation pattern of the triceps muscle to investigate the preferred triceps nerve for nerve transfer. Twenty-five cadaveric arms were dissected. The long head of the triceps received a single branch in 23 cases (92%) and double branches in 2 cases (8%) only. The medial head had a single branch in 22 cases (88%) and double branches in 3 cases (12%). The lateral head was the most bulky one and received more than one branch in all cases (100%), ranging from 2 to 5 branches. The transfer of the most proximal branch to the lateral head of the triceps seems to be the most preferred choice for deltoid muscle innervation.

## 1. Introduction

The triceps muscle is located in the posterior aspect of the arm. The muscle has three heads which eventually converge to be inserted as a single tendon. The muscle is supplied by branches of the radial nerve. There are multiple nerve branches supplying the triceps muscle, and this makes it very useful to be utilized for neurotization of the deltoid muscle without any significant functional impairment in patients with brachial plexus injury. Most authors utilize the nerve to the long head of triceps for nerve transfer [1]. However, no anatomical studies were done to investigate which triceps nerve would be preferred for the nerve transfer. This anatomical study was carried out to describe the innervation pattern of the triceps muscle and to investigate the preferred triceps nerve for nerve transfer.

## 2. Materials and Methods

Twenty-five cadaveric arms were dissected while the body is in supine position and the arm across the chest for the posterior approach of neurotization of the deltoid nerve. Twenty-two cadavers were utilized, 19 unilaterally and 3 bilaterally. All dissections were carried under loupe magnification. Since the nerve to the long head may arise from the axillary nerve [2], the relationship of the axillary nerve to the innervation of the long head was also examined.

## 3. Results

22 cadavers were dissected (3 bilaterally). Thirteen bodies were females and nine were males. All nerve branches to the three heads of the triceps arose from the radial nerve. The long head received a single branch in 23 cases (92%) and double branches in 2 cases (8%) only. In 6 cases, the single branch divided before entering the muscle. The first branch to the muscle arose at a distance of 2 to 10 cm from the humeral head (mean of 4 cm).

The medial head had a single branch in 22 cases (88%) and double branches in 3 cases (12%). The single branch arose in combination with the long head branch in one case (4%) and from the first branch of the lateral head in 2 cases (8%). The first branch to the muscle arose at a distance of 5 to 15 cm from the humeral head (mean of 7 cm).

**Figure 1:**
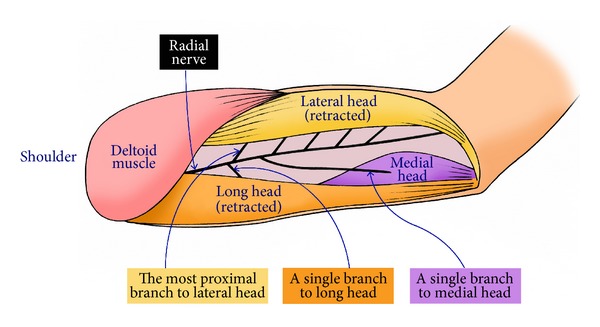
A diagram showing the innervation of the 3 heads of the triceps. Note that the lateral head always receives multiple branches (5 branches in this cadaver). In contrast, the long and medial heads usually receive a single branch.

**Figure 2:**
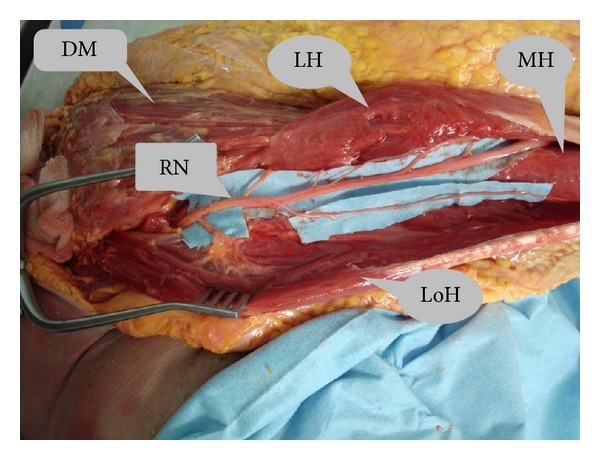
LH (lateral head of triceps), MH (medial head of triceps), LoH (long head of triceps), RN (radial nerve), and DM (deltoid muscle).

The lateral head was the most bulky one and received more than one branch in all cases (100%), ranging from 2 to 5 branches: 2 branches in 11 cases (44%), 3 branches in 7 cases (28%), 4 branches in 5 cases (20%), and 5 branches in 2 cases (8%). The first branch to the muscle arose 4 to 12 cm (mean of 6 cm) from the humeral head. Further branching of the branches is common. A representative diagram of a cadaveric specimen is shown in Figures 1 and 2, respectively.

## 4. Discussion

Anatomical text books state that the triceps muscle takes innervation from the radial nerve, but some studies showed that there is a variation in the nerve supply to the long head in which the innervation is by a branch from the axillary nerve instead [2]. In our study, all branches arose from the radial nerve. No anatomical study is available describing the pattern of innervation of the triceps heads and the number of branches for each head for suitability for nerve transfer. The use of the nerve to the long head is generally recommended in nerve transfer to the deltoid nerve [1, 3–7].

Our study showed that the long head is supplied by a single branch from the radial nerve in 92% of the cases, making it less preferred for utilization since the long head will be completely denervated in most cases. Furthermore, the nerve to this head rarely comes from the injured axillary nerve [2] and, hence, it may not be a suitable choice.

Our study showed that the use of the most proximal branch to the lateral head would be the most preferred for the following reasons. Firstly, the lateral head always received multiple nerve branches from the radial nerve and, hence, the head will not be denervated following the transection of the most proximal branch. Secondly, the most proximal branch arises from the radial nerve about 4 cm from the humeral head, and hence it can easily reach the deltoid nerve. Thirdly, all branches to the lateral head arise from the radial nerve with no anatomical variations as being arising from the injured axillary nerve.

## 5. Conclusion

The transfer of the most proximal branch to the lateral head of the triceps seems to be the most preferred choice for deltoid muscle innervation.
